# Determinants of Rabies Post-exposure Prophylaxis Drop-Out in the Region of San-Pedro, Côte d'Ivoire

**DOI:** 10.3389/fvets.2022.878886

**Published:** 2022-07-08

**Authors:** Rose Delima N'Guessan, Kathrin Heitz-Tokpa, Djedou Martin Amalaman, Sopi Mathilde Tetchi, Vessaly Kallo, Andrée Prisca Ndjoug Ndour, Govella Nicodem, Issiaka Koné, Katharina Kreppel, Bassirou Bonfoh

**Affiliations:** ^1^Sociology Department, Université Peleforo Gon Coulibaly, Korhogo, Côte d'Ivoire; ^2^Centre Suisse de Recherches Scientifiques en Côte d'Ivoire, Abidjan, Côte d'Ivoire; ^3^Institut National d'Hygiène Publique, Abidjan, Côte d'Ivoire; ^4^Direction des Services Vétérinaires, Abidjan, Côte d'Ivoire; ^5^Department of Microbiology, Immunology, and Infectious Pathology, Ecole Inter-Etats des Sciences et Médecine Vétérinaires de Dakar, Dakar, Senegal; ^6^Department of Environmental Health and Ecological Sciences, Ifakara Health Institute, Dar es-Salaam, Tanzania; ^7^Department of Sociology, Université Jean Lorougnon Guédé, Daloa, Côte d'Ivoire; ^8^Department of Life Sciences and Bioengineering, Nelson Mandela African Institution of Science and Technology, Arusha, Tanzania; ^9^Department of Public Health, Institute of Tropical Medicine, Antwerp, Belgium

**Keywords:** rabies, post-exposure prophylaxis, treatment completion, One Health, Côte d'Ivoire

## Abstract

**Abstract:**

Despite the fact that death from rabies is 100% preventable with a course of post-exposure prophylaxis (PEP) treatment, canine rabies still causes about 59,000 human deaths worldwide annually, half of which are occurring in Africa. In Côte d'Ivoire, rabies remains a threat partly due to the high drop-out rate of the life-saving human PEP treatment among people exposed to dog bites. Each year, half of the victims starting treatment, do not complete the course. The current study therefore assessed the determinants for drop-out of the life-saving treatment among people exposed to rabies in the department of San-Pedro in Côte d'Ivoire.

**Methods:**

A mixed-methods approach was used, including questionnaires, observation, individual interviews and focus group discussions, to gather socio-demographic and economic data from 235 participants about possible reasons for abandoning treatment. The study population consisted of patients and medical and veterinary health professionals who were selected using stratified sampling and purposive selection from a database available at the Rabies Center of San Pedro.

**Result:**

The drop-out of PEP treatment was related to perception bias and a habit of low attendance of health care and vaccination centers in the population. Quantitative analysis shows differences between rural and urban areas and an association with age when it comes to treatment completion. The dropout rate was most significant among patients who, in case of other illness, did not routinely see a doctor or go to vaccination centers. The rate of abandonment was higher among those who believed that dog-related injuries could be easily treated at home, and who believed that a person with rabies could be cured without completing the preventive treatment. Insufficient provision of health information on rabies and logistic constraints related to the practical organization of treatment, including the long distance to the anti-rabies center and weaknesses in the patient follow-up procedure, did not contribute to the completion of PEP.

**Conclusion:**

Established determinants for drop-out provide a framework for effective design and implementation of rabies control strategies to accelerate rabies deaths elimination efforts. In particular, access to PEP and community knowledge about rabies need to be improved and integrated in the health system and education system, respectively.

## Introduction

Canine rabies causes approximately 59,000 human deaths per year, primarily in Africa and Asia (1). This is of particular concern because deaths from rabies infection are 100% preventable with the timely administration of human post-exposure prophylaxis (PEP) in the early stages of exposure to rabies (2). Rabies prophylaxis consists of wound management and the administration of several doses of rabies vaccine (3) at the earliest moment after exposure, and before any symptoms appear, to avoid death and disability (see different treatment regimen below). However, achieving compliance remains a challenge in the African region, thus limiting rabies elimination efforts. In Côte d'Ivoire, according to the National Institute of Public Hygiene (INHP), the number of people exposed to rabies per year is approximately 12,000. Nearly 50% of those who begin prophylactic treatment in vaccination centers do not complete it (Republic of Côte d'Ivoire INHP, 2019), while only 12% of the canine population is vaccinated (4). Official deaths from human rabies are around 20 people per year. However, there are likely more unreported deaths in the communities (Republic of Côte d'Ivoire INHP, 2019). Most deaths occur in children under 15 years of age (50%) and the majority is in rural settings (60%) in the southwestern and northern parts of the country, with Bouaké and San-Pedro topping the list (Republic of Côte d'Ivoire INHP, 2018).

In San-Pedro, the post-exposure vaccine management is the responsibility of the INHP's rabies vaccination service. This service is concerned with non-adherence to the treatment protocol. Each year, nearly 500 cases of exposure are notified and treated by INHP-personnel, according to the national protocol of post-exposure care for rabies. The vaccination process is organized over a 21 or 28-day period with protocols ranging from 4 doses for the Zagreb Regimen to 5 doses for the Essen Regimen. Costs for the Zagreb protocol are 32000 FCFA (51,92 USD) and for Essen 40000 FCFA (64,33 USD). This means that each dose of vaccine is 8000 FCFA (12,86 USD) (Republic of Côte d'Ivoire INHP, 2019). As mentioned above, about half of the bite victims who start PEP, abandon the treatment after the first few doses, which may have fatal consequences. The underlying reasons for the poor adherence are unclear in the Ivorian context.

The abandonment of PEP is not unique to the African context. In China, for instance, despite the affordability of the treatment and the proximity of the center to the patients, victims consult the vaccination center too late, as PEP must be administered within a set time frame to avoid death or lifelong disability (5). A low level of knowledge about rabies in the population has been frequently cited as the main cause. Studies carried out in Côte d'Ivoire also mentioned that a lack of awareness leads bite victims to abandon the PEP before completion. In the city of Abidjan, studies identified lack of knowledge as a factor for patients abandoning treatment (6). However, the major factor for treatment abandonment is “a lack of financial means” (7, 8). In 2016, a strong partnership was formed through the Rage-GAVI project to estimate the burden of rabies in Côte d'Ivoire (4), which included national actors such as the National Institute of Public Hygiene (INHP), the Ministry of Livestock and Fisheries (MIRAH), the Swiss Center of Scientific Research in Côte d'Ivoire (CSRS-CI), as well as international partners [Global Alliance for Vaccines and Immunization (GAVI) and the Swiss Tropical and Public Health Institute (Swiss TPH)].

The research to estimate rabies cases was carried out in the areas of Bouaké and San-Pedro, two cities with a high rabies burden (4, 8, 9). At the time, the Zagreb and Essen treatment protocols were in use. As it is generally known that costs prevent victims from completing the vaccination regime (10), the rabies centers in San-Pedro and Bouaké offered the new Thai Red Cross vaccination protocol free of charge alongside the traditional protocols, during the project period. This was expected to reduce the dropout rate (11). The Thai Red Cross protocol requires four appointments over a period of 28 days. Each time two doses are administered. In comparison, the existing Zagreb and Essen protocols, require 3 visits to administer 4 doses and 5 visits for 5 doses, respectively.

A the end of the intervention, the rate of non-adherence had decreased for all three protocols, reducing the dropout rate from 50 to 35% (9, 11). A large majority (78%) of the patients chose the Thai Red Cross protocol offered free of charge. Astonishingly however, the dropout rate, was significantly higher among those who used the free vaccine protocol than among those who chose a fee-based protocol.

In the face of the dropout that occurred during the period when the vaccine was free in San Pedro, the results of previous studies alone, the majority of which fall within the field of epidemiology, are not sufficient to explain the lack of adherence to treatment. The underlying reasons for low adherence to PEP in San Pedro appear to be differences in the way patients relate to exposure and use of the health center in case of illness in general.

Therefore, a more holistic approach is needed to understand non-adherence to PEP. Our study in social science, which is part of a long-term research partnership between human and animal health and a transdisciplinary One Health approach, therefore aims to describe and analyze the social determinants of non-adherence to PEP treatment that occurred during the period of free health care in San Pedro.

## Methodology

### Site and Period of the Study

**Figure 1:**
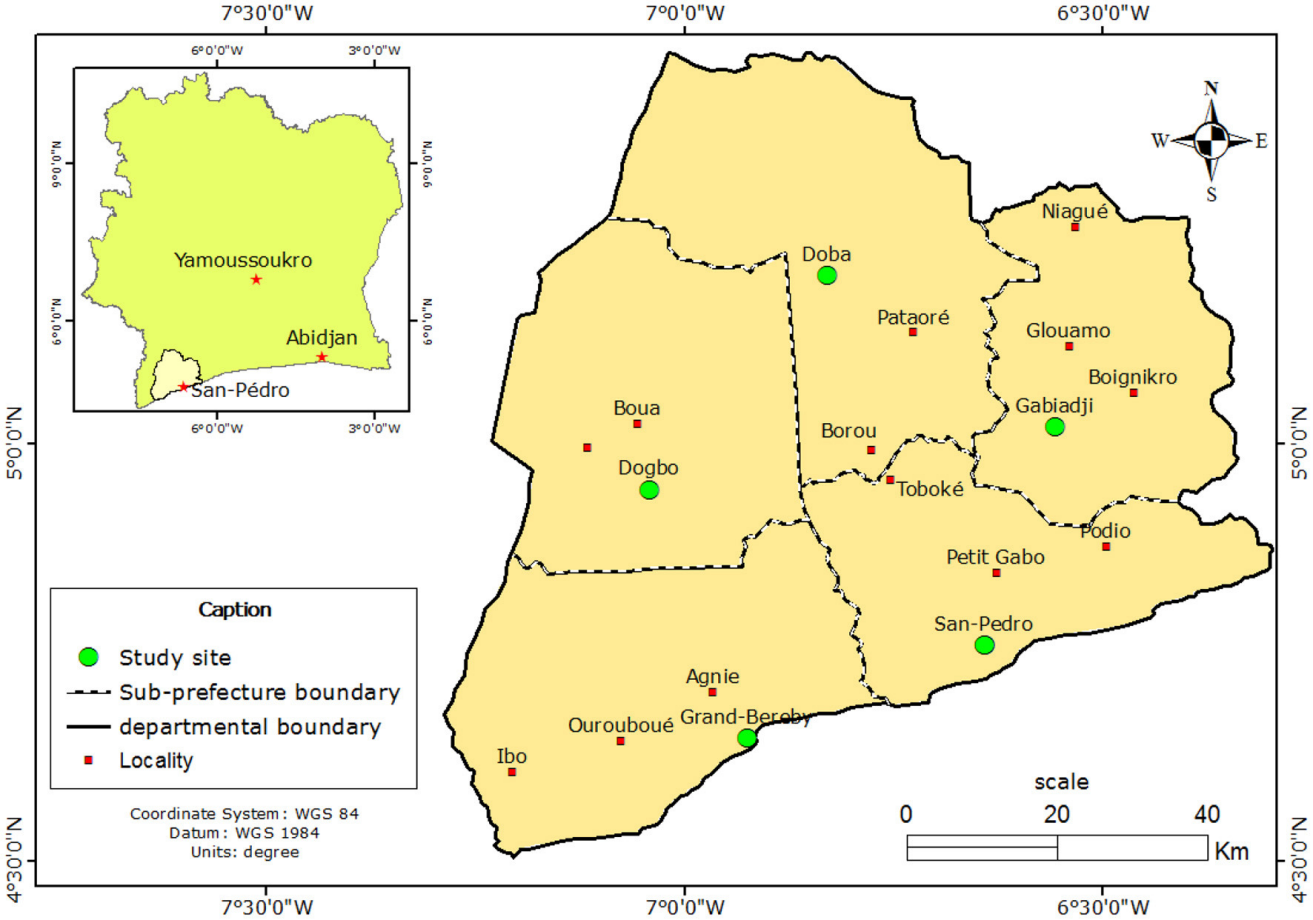
Study site (*N* = 5), cross-sectional study conducted from 14 February to 28 April 2019 in the department of San-Pedro, Côte d'Ivoire.

The social science study took place from February to April 2019 in the rural and urban areas of the five (5) sub-prefectures of the San-Pedro Department, namely San-Pedro, Grand-Béréby, Dogbo, Doba and Gabiadji. San-Pedro is the fourth largest city of Côte d'Ivoire and located in the extreme southwest of the country, bordering Liberia to the west and the Gulf of Guinea to the south. The San-Pedro Department belongs to the Bas-Sassandra district (Figure 1). The department covers an area of 7,072 km^2^, with an estimated population of 562,000 inhabitants, of which 140,000 live in towns and 422,000 in rural areas.

San-Pedro has port activity, agriculture, agro-industry and tourism. The inhabitants are also involved in small and medium-sized enterprises, of which trade and handicrafts are the main activities. This locality is governed by state authorities and traditional authorities such as village chiefs, notables, village elders and religious authorities.

In the area rabies is endemic and has a high prevalence. At the time of the study the department had approximately 800 dogs according to official data with an estimated dog vaccination coverage of only 7% (4). The main activities with which these animals are associated are guarding and hunting. Although many dogs have known owners, many stray dogs are left on the streets. Attached to the sub-prefecture of San-Pedro, a rabies center services animal bite and scratch victims from all five sub-prefectures.

### Type of Study and Tools for Data Collection

This cross-sectional study collected both qualitative and quantitative data. For the quantitative component, a questionnaire was developed to collect data on socio-demographic and socio-economic characteristics of the bite victim. Questions were answered by the victims themselves or by their guardian at the health center. Other data collected included information on the etiological understanding of rabies, vaccine prevention, therapeutic itineraries, and perceived challenges of vaccine management. Additional qualitative data were collected through semi-structured individual and group interviews. Interview guides were developed and administrated to patients or their guardians and to health professionals. The objective of the qualitative interviews was to discuss aspects in depth, including the motivations for therapeutic behavior, that the questionnaire alone did not cover.

### Target Population

The study covered two types of stakeholders: community members and professionals in the human and animal health sectors. People in the first category were composed of dog bite and scratch victims and their guardians who had visited the rabies center between June 2017 and March 2018, during the free vaccination period. During that period, 35% of patients did not complete their treatment.

People in the second category were composed of personnel from the National Institute of Public Hygiene (INHP), the Ministry of Animal Resources and Fisheries, the local committee for rabies control, and the public and private veterinary services. With them, we discussed how management and control of rabies in the department were organized.

### Sampling Method and Data Collected

Respondents for the questionnaire survey were selected using a stratified random sampling technique to select a representative sample. Data for 409 patients (and their guardians) was available at the INHP rabies center to which we had access through the consent of the GAVI project. After sample size estimation (12) for quantitative survey studies, a sample size of 199 victims was determined. Some information on socio-demographic characteristics of the patients was already included in the case files such as sex, age, place of residence, occupation, vaccine type used, number of visits to the center and contact details. Based on this information, we used proportional stratified sampling as explained by N'Da (13) to then randomly select the 199 individuals needed for the study. The stratification process considered place of residence, sex, social class, and type of vaccine used. In cases where patients could not be contacted, they were replaced with other patients with the same characteristics.

During the recruitment process, which included the examination of the victims' files at the rabies center to select a sample representative of the whole pool of patients, we faced several challenges. Since this study commenced 2 years after the project, contact details for several selected patients had changed. Furthermore, patients who abandoned treatment turned out to be more likely to decline to participate. Replacements were made from the original database each time, until the pool of drop-out patients was exhausted. Thereafter the required sample size was reached by including victims who completed the treatment. The lead author visited the selected households to administer the questionnaire using the software “ODK collect” on a tablet (http://dl.acm.org/citation.cfm?id=2369236; https://getodk.org/). On a few occasions, when the respondents did not want to meet in person, the questionnaire was administered via phone.

Less than a third (28%) of the respondents were victims themselves, due to the fact that most dog bite victims are underage and interviews were therefore conducted with their parents/guardian.

Our respondents were composed of women and men with different backgrounds, religious faiths, educational levels, marital status, ethno-national identities, from different household types and engaged in different economic activities.

To gain further insights into the obstacles victims face to complete the treatment, qualitative methods were used to collect data on health seeking and medical treatment habits in general, including treatment of bite wounds and access and affordability of care (14). Informants for the individual interviews were selected from both categories, drop-outs and people who had completed the treatment, using a convenience sampling technique. If they consented, the interview was held immediately after the questionnaire. After 25 individual interviews with patients or their guardians, including 15 who had abandoned and 10 who had completed their treatment, the interviews were stopped, as saturation was reached. From the institutional side, two human health professionals, two animal health professionals and a representative of the local rabies control committee in San-Pedro were interviewed. The interview guide covered questions about awareness campaigns and care practices, as well as any difficulties encountered for both activities.

Two focus group discussions (FGD) with six participants each were conducted at the rabies center of San Pedro to deepen the understanding of reasons for children to abandon treatment: One FGD was conducted with the parents or guardians who had accompanied the child victim to the meeting, the second FGD—held in parallel—was conducted with the children themselves. Bite victims were selected after the administration of the questionnaire if they were willing to participate. The objective of the FGDs was to assess the children's knowledge of rabies and to compare their level of knowledge to their parents'.

### Ethical Dimension and Conduct of the Study

This study was covered by the ethical approval of the GAVI Project: ethical approval (N/Ref: 072/MSHP/CNERkp) from the National Ethics Committee of Côte d'Ivoire. The agreement of the Northwest and Central Ethics Committee of Switzerland was also obtained [Ethics Committee of North Western and Central Switzerland (EKNZ) Basec 2016–00,220]. At the research site, we sought survey authorization from the Ministry of Higher Education and Scientific Research, the INHP, the Directorate of Veterinary Services, the regional prefect and the sub-prefects. During our visits to households, we presented these authorizations and gave an oral explanation of the purpose of our presence before the start of the interviews in order to obtain the participants' informed oral consent. Thus, only those who agreed were interviewed.

### Data Analysis

The quantitative data was analyzed using STATA 14.2 (StataCorp; College Station, USA) and R software version 4.1.2 (Core-Team. R: A language and environment for statistical computing. *R Foundation for Statistical Computing, Vienna, Austria*, 2019).

Descriptive analysis was carried out with different socio-demographic characteristics related to the respondents. This was followed by general linear regression (GLM) modeling with binomial distribution to investigate associations between treatment completion (yes, no) and variables on socio-demographics of the victim. Models were selected based on backward elimination from an initial null model that included all variables. A likelihood ratio test (LRT) was used to test each variable and remove those with the highest *p-*value at each iteration from the maximal model until only statistically significant terms remained. Model fit was assessed by plotting the coefficients and inspecting the residuals against the fitted values. To determine the influence of statistically significant variables, LRTs were used. The package “MASS” was used in R (15).

Analysis looked at the association between treatment completion as dependent variable, and the characteristics of the bite victim as independent variables. These explanatory variables were “sex”, “age bracket”, “education level”, “religion” and urban or rural “zone”, “district” as well as “number of people in the household” and household “income”.

For the qualitative data, content analysis was conducted. In an iterative inductive process, the transcribed interviews were coded, and themes identified in relation to the research questions (16). We have selected quotations to deepen and illustrate aspects of the quantitative results. To analyze and interpret the findings, we drew on the following theoretical texts: the concept of cultural capital (17), which stipulates that practices of care are influenced by all the know-how available to the individual as a result of the socialization at the family and environmental level and the conceptual framework five A's of access (availability, accessibility, affordability, adequacy, acceptability) for access to care (14) with a particular focus on accessibility and affordability of care.

## Results

### Socio-Demographic and Economic Characteristics of Patients and Respondents

A total of 199 individuals participated in the study from all 5 sub-prefectures of the San-Pedro department in Côte d'Ivoire. A summary of the respondents' socio-demographic characteristics is shown in Table 1. Most suspected rabies patients were male children (75.3%) of which 65.2% were in school. The average age was 10 years and as a result, most of our respondents (72.3%) were the guardians of the underage bite victims. Only 27.6% of those interviewed were themselves victims (adults). Respondents' education levels were as follows: primary school education level (29.8%), secondary school education level (29.8%), higher school education level (20.2%), no school education level (20.2%). The average monthly household income was 100 000 FCFA (160.84 USD). Participants resided in the sub-prefectures of San-Pedro (83.9%), Gabiadji (6%), Grand-Béréby (5%) and Doba (2%). The majority of participants lived in urban areas (85.4%) compared to 14.5% from rural areas. They practiced Christianity (69.4%), Islam (21.6%), other religions (4.5%) or no religion (4.5%). Treatment completion was 81.9 percent and drop-out rate was 18.1 percent.

**Table 1:** Summary of the socio-demographic characteristics of victims only and of all respondents.

**Socio-demographic characteristics**		**Frequency**	**Percentage (%)**
Sex of victims	Female	49	24.62
	Male	150	75.38
Education of victims	No formal education	23	11.56
	Primary	130	65.33
	Secondary	38	19.10
	Higher	8	4.02
Status of the respondents	Victims	55	27.64
	Parents	144	72.36
Education of respondents	No formal education	40	20.20
	Primary	59	29.80
	Secondary	59	29.80
	Higher	40	20.20
Place of residence of victims and respondents	San-Pedro	173	86.93
	Gabiadji	12	6.03
	Grand-Béréby	10	5.03
	Doba	4	2.01
Zone of victims and respondents	Urban	170	85.43
	Rural	29	14.57
Religion of victims and respondents	Christian	138	69.35
	Muslim	43	21.61
	None	9	4.52
	Other	9	4.52
Victim's vaccine completion	Complete	163	81.91
	Drop-out	36	18.09
			

### Factors Associated With Treatment Drop-Out

#### Patient Profile

The first model of our analysis showed that only two characteristics of the victim are associated with treatment completion and discontinuation: age and zone of residence (rural or urban). The odds of abandoning treatment were significantly higher when the victim was between 25 and 29 years of age. Moreover, urban residents were more likely to complete the treatment than their rural counterparts (Tables 2, 3). According to the qualitative data, this may be due to distance (see below).

**Table 2:** Model results of association between post-exposure rabies treatment drop-out and socio-demographic and economic characteristics of rabies victims in rural and urban areas with 95% Confidence Interval (CI) and *p-*value.

**Variable**	**Odds Ratio**	**2.5% CI**	**97.5% CI**	***p-*value**
Model 1*
**Age (years)**
0–10	1	NA	NA	NA
11–15	16.68	0.541	46.661.399	0.345
16–18	0.743	0.039	44.377.313	0.786
19–25	33.801.620	0.717	148.844.944	0.107
26–29	67.998.961	159.181.655	308.483.962	0.009^∞^
≥30	0.621	0.16447036	18.871.908	0.434
**Zone**
Rural	1	NA	NA	NA
Urban	0.3653479	0.14204669	0.9753399	0.03857*

^∞^Significant p-value at the 5% level. *Modeling the victims' characteristics associated with treatment abandonment. NA, not applicable.

**Table 3:** Likelihood ratio test χ^2^ values of all significant variables in the final generalized linear model on the probability of abandoning treatment.

**Variable**	**χ^2^**	***p-*value**
Zone (Urban area)	4.032	<0.044
**Age (years)**
26–29	11.1	<0.049

#### Knowledge of Rabies Etiology Among Respondents and Patients

Data from the questionnaire showed that a total of 94% of respondents had heard of rabies as a dog disease while 6% did not know it was a disease affecting canines. The disease was known under different terms and in general, people who lived in urban areas and had received formal education called rabies primarily “rabies”. Those who lived in rural areas and had little formal education called rabies “canine disease” or “canine madness”, which is often a direct translation from local languages (Baoulé, Malinké) into French.

Respondents generally knew that humans could contract rabies and dog bites were identified as the main mode of transmission (74.7%). They mentioned aggression (40%), drooling, hair loss (30%), wasting (10%) and excessive barking (15%) as the main symptoms of rabies in dogs. In humans, symptoms were completely unknown (58.8%) or poorly known (associated with diarrhea, drooling, wasting, hair loss and vomiting, 27.7%; aggression, 13.4%).

As far as knowledge about the severity of rabies is concerned, the study found that 81.9% of respondents did not consider it to be a serious disease, neither for humans nor animals. While 27% said that it is impossible to cure a dog with rabies, 20.6% said the opposite. The remaining 52% did not know whether a dog could be cured of rabies or not. For humans, 29.71% were certain that rabies could not be cured, while almost half of respondents (43.43%) were certain that a rabies patient could be cured. The remaining 26.86% were unable to respond due to lack of information.

The interviews revealed little information about the knowledge on the signs of the disease, but some identified rabies as a dog disease, not a human disease. However, they had difficulties giving accurate descriptions about symptoms of the disease. The focus group of patients' parents on knowledge of rabies etiology showed low knowledge of the severity of rabies. Respondents seemed to be aware of dogs as vectors of the disease, but were not aware of the outcome of the disease.

There was an indication that people from urban areas had more knowledge or access to knowledge regarding the urgency to seek treatment as statements show:

Q1: “*Well, people said, especially my neighbor, to quickly go with him to the hospital that if I waited, he was going to be like the dog, too. Otherwise, I do not know much about the disease.”* (Mother of non-compliant patient, 40s, no formal education, urban resident, IDI).

Q2: “*Well, what I know is that if a dog bites you, it can make you sick. If you don't go to the hospital to get antibiotics or a tetanus shot, it can give you tetanus. But, if you go and get at least one vaccine, you are safe.”* (Father of non-compliant patient, 40s, secondary school level, urban resident, FGD).

The focus group conducted with exposed children revealed that they had a much higher level of knowledge on rabies than adults. More than half of children had an accurate description of rabies symptoms, compared to adult patients and parents from another FGD.

Three-thirds of children identified rabies as a disease transmitted by dogs and cats and were clear about the mode of transmission and the consequences of infection.

Q3: “*[…] dogs transmit rabies. When they bite someone, if they don't go and get vaccinated there, one day they can go crazy and act like a dog.”* (Patient, 9 years, primary school level, urban resident, FGD).

#### Perception Related to Rabies Exposure and Post-exposure Treatment

Interviews and data from focus groups revealed different types of perceptions of dog attacks and vaccine treatments. The first perception was commonly expressed by parents of non-compliant patients living in urban areas whose education ranged from non-formal to secondary education. These respondents perceived dog bites and scratches as ordinary injuries that could easily be treated at home. The attack of a person by a dog was presented as a natural occurrence that had no cultural significance. Some participants compared a dog bite to an insect bite, which they felt should not be interpreted in any other way, nor associated with the spiritual world.

Q4: “*When a dog bites a person, it is not something spiritual. Otherwise, people will say that when a mosquito bites you it is a curse or a blessing. In any case, it doesn't mean anything.”* (Compliant patient, 30s, secondary school level, urban resident, IDI).

Other participants interpreted dog bites as a mystical act. For instance, they considered dog attacks as an omen. An omen is considered positive, when the aggression has a positive connotation in favor of the aggressed (delivery of a spell, good news in progress) or negative when the aggression is not in his or her favor (a spell cast on the victim, an upcoming misfortune etc.). This perception of a dog aggression as a mystical act led study participants to say that they were destined to suffer dog aggression.

Q5: “*When a dog bites you, it can be a sign of misfortune or blessing. So, if it bites you, you must pray to God. A dog does not bite everyone and not in vain.”* (Father of non-compliant patient, 40s, primary school level, rural resident, IDI).

Nevertheless, whether the respondents considered dog attack wounds as ordinary wounds or as an omen, they went to the health center for the same reason, namely wound care. In the case of both groups, care was more focused on managing the wounds for prompt healing than on any illness that the bite may cause. People from both groups did not, however, deny that being bitten by a dog is a real danger to the victims. The role of prophylactic treatment was described as supplementary wound care:

Q6: “*We were going to the hospital to treat the wound. But his wound healed before the vaccine was finished. So, we didn't go to finish the vaccine anymore.”* (Father of non-compliant patient, 40s, secondary school level, urban resident, IDI).

### Treatment Habits

The data of patients' therapeutic itineraries after exposures revealed a plurality of itineraries. Almost half of the patients had used the vaccination center as their first point of contact for treatment. However, only 10% went immediately. The rest (90%), waited for hours, days, and weeks without seeking treatment before going to the rabies center. The reasons for this delay presented three types of health care seeking behavior. Almost three quarters decided to seek treatment at home first, before going to the rabies center (74%), while 25% used community health centers first. The remaining 1% used traditional healers.

Q7: “*We went late to the vaccination center because we were treating her at home. As the wound became bigger, we went to see the local doctor and he asked us to quickly go to the place where they treat dog bites, because if we don't go it's not good.”* (Mother of non-compliant patient, 50s, no formal education, urban resident, IDI).

However, nearly half of the patients who used home treatments, also received vaccination doses at the same time and almost all patients (80%) practiced home care at one point for healing. Patients living in rural areas used mostly a herb, “*sékou touré*” *(eupatorium odoratum L*.), that usually grows on the side of the road, mostly in villages, and cassava leaves *(manihot esculenta)* as well as the cassava itself for wound care. “Black powder” concocted by traditional practitioners was purchased and used with hot water. In contrast to rural residents, urban residents used disinfectants, salt and bleach, as well as tablets purchased from local shopkeepers. Remedies varied by area of residence. However, the use of hot water remained common practice. The logic associated with this use of complementary or alternative care was based on trust placed in these remedies and the experiences of use.

Q8: “*Plants are medicines. One plant can cure several diseases. Now, if you know […] the plants, why waste all your time in the hospital instead of healing yourself [with the plants] […] It's for the wound. These are things that everyone knows. In all my life, I have never seen hot water aggravate a wound. On the contrary, our parents applied it on us and we didn't die.”* (Father of non-compliant patient, 50s, high school level, rural resident, IDI).

Q9: “*In any case, that's how it is with us. Before we run to [the health facilities], we take care of ourselves first. Otherwise, why should our parents have taught us these things?”* (Mother of compliant patient, 30s, secondary school level, rural resident, IDI).

Hence, most respondents showed some attachment to complementary treatments such as self-medication, street care or traditional treatments when cases of illness occurred. For the care of certain conditions such as malaria, some respondents did not necessarily consult a health center. Instead, they used traditional treatments and bought drugs in the informal market. In general, patients did not associate wound management with vaccination centers, even though patients were used to receiving one injection at health centers at the beginning of any wound treatment to prevent tetanus.

An important finding of our investigation of health care seeking habits is that the majority of the patients who dropped out from the vaccine treatment, generally only used health centers for what they perceived as serious diseases. People would go to the health center right at the onset of an illness for diagnosis or at the later stage of a disease for intensive care when other treatments had failed. just over half of the participants reporeted health centers as their preferred place for emergency treatment (55.7%).

### Organization of Rabies Vaccination

#### Accessibility and Transportation Costs

An important observed factor of access to PEP is the location of the immunization center. It is located centrally in the regional capital, San-Pedro, and patients are distributed throughout the department of San-Pedro and its five sub-prefectures (San-Pedro, Béréby, Dogbo, Gabiadji, and Doba). The distance between San-Pedro and Dogbo is approximately 102 km, 55 km for Grand-Béréby, 104 km for Doba and 45 km for Gabiadji. Many of the villages of the mentioned sub-prefectures are located even further away from San-Pedro than the main town and are often in remote areas that are difficult to access.

During visits to the vaccination center, most patients used intercity minibuses communal cabs or motorcycles. The duration of the trips often varied between 1 h, 2 h, half a day or even a whole day, depending on the area of residence, the means of mobility and the state of the roads. However, particularly in rural areas, that roads were impassable during rainy season.

The results on geographic accessibility show that for the population, costs and exhaustion linked to the distance constituted an important obstacle for patients to access care. For most patients from remote areas, the duration of the treatment and frequency of visits requested were a hindrance to care completion.

The average monthly household income was 100000 FCFA (160.84 USD) for four people per household. The cost of travel to and from the vaccination center per individual was estimated at between 0.85 USD and 1.70 USD for the neighborhoods closest to the vaccination center and about double for the more distant neighborhoods. Individuals from remote areas paid between 5.10 USD and 20.40 USD. These travel costs were doubled if the patient had to be accompanied, which was often the case due to the high number of underage bite victims. In general, patients were accompanied by at least one relative when visiting the center.

This means that even for people who live close, transportation costs correspond to the daily income of one person. For those living further away however, the return-fare to the rabies center for the patient and a person accompanying the bite victim amount to a monthly income of one person. We can conclude that transportation costs and indirect costs linked to the loss of income played an important role in the decision to discontinue the treatment.

#### Pre-exposure Management of Rabies

Data collected from the patient and parents of patient's interviews revealed that most respondents (94%) had heard about rabies through various channels. The most frequently mentioned was at school. Respondents reported having received information about rabies during their primary and secondary school years. They also said that they had not attended any sensitization events for the prevention of rabies, except from some information received during their visits to the rabies center. They had hardly ever attended an awareness campaign to inform themselves about the seriousness of dog bites.

The data collected with health professionals revealed difficulties in the management of rabies in San-Pedro. They identified the problem of funds as a constraint to the implementation of general awareness activities for the population. Although outreach campaigns are organized, at least during the Word Rabies Day, they are generally directed at community leaders who in turn are responsible for sharing the information received with their communities. How this is done, with what means and to what extent is unclear.

#### Patient Follow-Up in the Post-exposure Management

As mentioned above, the GAVI project offered free vaccination from June 2017 to March 2018. However, our results showed that 95.98% of the people who visited the rabies center for treatment had not been informed in advance, i.e., before their arrival at the center, of the availability of free vaccine. This suggests that the project has not managed to communicate successfully to the population on the availability of the free vaccine.

Q10: “*I didn't know there was a free vaccine. It was when I arrived that I knew. So I agreed to use it, because I didn't have much money.”* (Patient, 20s, Primary school level, male, urban resident, IDI).

Furthermore, asked about their vaccination sessions at the rabies center, half of the patients reported that they had not been further sensitized about rabies, its severity or the need to continue their treatment to term (52%).

Q11: “*They didn't explain anything about rabies. When I arrived, he told me the price of the vaccine and then he filled out some paperwork and then when he vaccinated me, he gave me my booklet and told me to come back in a week.”* (Patient, primary school level, male, 30s, urban resident, IDI).

Regarding the management of patient's follow-up appointments for their next doses, the vaccination center keeps records in a patient file in which each patient's date of the follow-up visit is recorded. However, this information is not systematically used to follow up on patients. Patients are not contacted when they do not attend the scheduled appointments. Health workers informed us that the rabies center in San-Pedro lacks a social service that exists in Abidjan, and which is dedicated to follow-up and any social aspect of patient care. Hence, a follow-up system or device to remind patients of their upcoming appointments is lacking in the rabies center of San-Pedro.

## Discussion

Our research revealed perception bias of the severity of rabies and a habitual low attendance of health care and vaccination centers of the population. Quantitative analysis suggests differences between patients from rural and urban areas regarding treatment completion. Drop-out was also higher among those who believed that dog-related injuries could be easily treated at home and who believed that a person with rabies could be cured without completing the preventive treatment. We also found a lack of knowledge on rabies and challenges in the practical organization of treatment.

Our study shows that offering the vaccine free of charge has helped to bring the rate of PEP treatment abandonment down from 50 to 35%. This is in line with findings of a study in Chad which found the same effect when comparing dog owner-charged with free vaccination campaigns (18, 19). Despite some challenges of the implementation of the campaign in Côte d'Ivoire, results strongly suggest that free PEP alone does not ensure compliance.

This relates partly to affordability of the treatment which is not just related to the direct costs of the vaccine. Indirect cost of transportation, loss of study time and income also must be taken into consideration (10, 14). For most of the non-observant patients at school age, repeated absence at school was perceived as awkward and those working in the informal sector felt that they could not afford more days without income. How costs may be shared between the patient and the public health system has to be further reflected upon and goes beyond the scope of this article.

A holistic perspective when looking into health care seeking behavior in context of rabies treatment completion therefore, seems to be a more suited approach. This will enhance our understanding of the populations' care practices in case of dog bites that expose victims to rabies.

As a first step, we have to look at dog bites as wounds and ask how such wounds are generally treated. As the data show, health centers are only consulted when people judge the situation as severe. In our case, according to the study participants, a bite wound by a dog is often not perceived as severe. This has to do with a lack of knowledge, which corresponds to findings from studies from Côte d'Ivoire (7, 20) and elsewhere in Africa (21–23) and beyond (5). Yet our study population were all people who consulted the rabies center for care, where they received a consultation from health workers. Recent studies from Chad revealed that the knowledge of health workers needs to be improved (24) and for Côte d'Ivoire we can conclude that the way counseling is provided has to be improved. Furthermore, the qualitative data from both the victims and the health personnel also showed that the follow-up system has to be strengthened. According to the health workers, the rabies center in San Pedro lacks social workers or a system assisting with patient follow up after they received their first dose. In view of the dropout rate (35%) despite the free vaccine, such a service may be crucial to improve treatment completion.

However, the whole chain of events regarding wound treatment and follow up care should be considered. Numerous studies from the field of medical anthropology have highlighted that care practices in Africa are best conceptualized as a therapeutic continuum with different medical options of which the patient takes different combinations (25). For instance, as observed in our study, victims may first treat the bite wound at home before seeking medical care at the health centers, which is absolutely necessary to combat the rabies pathology, and later complement the treatment of the wound with herbal baths at home.

The sample of this study was based on patients from the list of the rabies care center, hence excluded people who had exclusively consulted traditional healers and only used home remedies. It is known from studies estimating the burden of disease for rabies, that many cases go unreported because they are treated at home (4, 25, 26).

During field work, a man said that his wife and son must have died of rabies after having been bitten by their rabid dog who left their house afterwards. For years, he had thought it had been witchcraft and consulted a witchdoctor to protect himself and the younger children (Rose N'Guessan, personal communication, San Pedro, 2020). Such and similar cases highlight that many deaths are never reported. It was by serendipity, that such a case was reported.

To save lives, it is crucial that the awareness of rabies is raised in a tailor-made manner in which cultural barriers are addressed. Moreover, traditional healers need to be sensitized. Because of shortcomings of the health service provision, part of the population is used to rely on traditional healers and their advice is crucial. Rather than seeing them as obstacles, if sensitized, they may help to transfer bite victims in good time to health facilities where patients can receive PEP.

The need to actively improve the connection between health centers and the communities they serve is evidenced by the fact that many bite victims are hard to reach, not only for health care provision, but also for research. As mentioned in the methods section, many victims who had abandoned the treatment were no longer reachable 2 years after the end of the campaign on the phone number they had provided. During this period, the Ivorian state requested all numbers to be registered with ID cards for security reasons, and if that did not happen, the number was blocked. This and other reasons linked to difficulties of replacing a lost or stolen phone due to poverty, have made contacting hard to reach people, difficult. Without the means to follow up on patients, health workers and researchers cannot establish if treatment abandonment has had fatal consequences. This could be addressed by registering a second phone number from a relative who has not been bitten by the dog. Furthermore, some of the patients that had dropped out, often refused to participate in the study. Because they had not completed the treatment, some may have feared some kind of reprimand for non-compliance.

Dog bites and scratches are treated like other ordinary wounds at home, and more than half of interviewed patients believe they can be easily treated with home remedies. More than half believe that if a patient does not follow their preventive treatment correctly and is later found to have rabies, they can still be treated and cured. This way of thinking leads to lack of interest in or neglect of preventive treatment.

Regarding the lack of knowledge that emerged as a factor of non-adherence in the analysis of our data, the literature shows that the therapeutic practices observed in patients are a reflection of what they know about their condition as a sick person or as a person at risk (27). We observed that the less respondents know that rabies is incurable, the more they abandon preventive treatment.

More than half of the respondents (72.4%) were parents of patients, most of whom had heard about rabies during their primary school years and had an educational level between primary and secondary education. The average age of these parents was 45 years. These factors accounted for “vague” and “erroneous” knowledge about rabies, which influenced their treatment practices. Purchasing power and the decision to treat which does not necessarily belong to the exposed person, forces children to abandon treatment even when they have good knowledge of the disease (28). Representatives of the rabies committee in San Pedro reported that a child who was bitten by a dog in one of the districts had asked her parents to be taken to a health facility, as she had been sensitized at school, but the parents took her to a traditional healer and she later died (representative of rabies committee in San Pedro, transdisciplinary workshop, Abidjan, 2020). That children often do not have a say in the treatment they receive has also been shown in Tanzania in a similar context (29). This shows that sensitization campaigns at schools are not sufficient to make sure all bite victims benefit from PEP. From our data, it seems that the sensitization strategy does not contribute effectively enough to making the population aware of the dangers associated with dog bites in order to increase adherence to vaccination.

In Côte d'Ivoire, a study in Abidjan also mentioned this factor of non-adherence to prophylaxis (6). Studies in Abidjan and in San-Pedro and Bouaké, described the lack of knowledge of rabies as an important factor shaping people's attitudes and practices (7, 20). In Abidjan it was found that low knowledge about rabies correlated with non-vaccination of dogs by household heads in Abobo (30). In Burkina Faso, a study showed that rabies was not known to most of the respondents (31). Moreover, in a Cameroonian study with a predominantly dog-owning population, rabies was attributed to a sexually transmitted disease (32), which did not contribute to the use of rabies vaccine by dog owners. In China, the lack of knowledge about rabies was noted as the main reason for inappropriate wound treatment and delayed PEP (5, 33).

Yet this was not found in all studies. Research conducted in Abidjan on the abandonment of PEP for rabies showed successively that the treatment drop-out was linked to the dog owners' refusal to cover the costs of the patients' treatment, as well as the lack of financial means for the purchase of vaccines (7, 8). Costs also seemed important in our study, particularly indirect ones, but our results show that the role of financial means may have been overestimated in other studies. Our study was conducted in a period during which PEP was offered for free. This allowed us to see a more nuanced picture about causes contributing to the dropout rates, where the lack of knowledge and general treatment habits of patients together rank higher. However, our study which was not conducted in the countries capital, also laid bare the difficulties linked to accessing health facilities from remote areas (accessibility) and afford PEP for rural populations (affordability) (14).

Our analyses show that low attendance at health and vaccination centers in patients' habits was a determining factor in non-adherence to prophylactic treatment. It was observed that patients abandoned the treatment, because they rarely attended vaccination and health centers when they had other types of diseases. In case of illness, and depending on the perceived risks of severity, as well as the habit of care by assimilation of the disease or exposure, they prefer to resort first to traditional treatments or self-medication. Some use health centers only once when they are ill to validate the diagnostic hypothesis before returning for traditional or self-medication treatment alone or in addition to the treatment recommended by the physicians. For others, return to health centers is only favored when the disease worsens, and they no longer have other options. The course of the disease guides and defines the therapeutic resources used (34). Self-medication and traditional treatments are very common in Africa (25, 35). In the absence of a well-functioning public health system with trained medical personnel, medical pluralism that alternative treatments in is parallel and intertwined health care practices are likely to persist. Therefore, underserved and isolated communities heavily rely on traditional healers and self-medication. As a result, health centers are only visited when traditional treatment fails and when there is no other option. In the case of rabies, this is, unfortunately, always too late.

This weight of therapeutic habits among patients is strongly linked to their level of health education. In our context, it therefore highlights the importance of therapeutic habit in the intention to adopt a new desired behavior, which in this case is compliance with prophylactic treatment.

The therapeutic education acquired by patients in their experience, either through the family, the groups to which they belong and the environment, is reproduced when cases of disease occur (17), especially when they have no knowledge of the diseases. Thus, the low attendance of health and vaccination centers in the patients' habits in general determines the non-compliance with prophylactic treatment. Our results also showed that the age group of 19–25-year-olds has a higher risk of not completing treatment which could be related to their lack of experience as young adults.

Results reveal a centralization of rabies treatment that proved to be a hindrance to PEP. Due to the requirements of conservation of the vaccines and the specific character of their administration, patients were therefore obliged to travel long distances to the health center on routes that were difficult to navigate at least four times within a period of 28 days. Thus, the long distances traveled by patients due to poor track conditions and unavailability of transportation led to the retraction of their vaccination decision. These factors, combined with the cost of transportation, which was sometimes considered excessive, especially since patients were always accompanied by at least one parent and belonged to households with a monthly income of only 160.84 USD, influenced the decision whether to use the center. Thus, the lower social categories will consider proximity in their choice of health center, unlike those who have a more comfortable social position. In the abandonment of rabies PEP and animal vaccination, a study conducted on the determinants of rabies post-exposure prophylaxis abandonment presented this factor as well (36). It was found that populations who resided far from the vaccination center took significantly longer to reach the vaccination center to start treatment. It was showed how accessibility and lower economic status were the main factors associated with delayed initiation of prophylaxis for rabies prevention (37, 38).

As far as the limitations of the current study are concerned, it is important to note that the initially desired sample size of 209 people was not reached. Only 199 people could be interviewed, which gave us 95% of our initial sample. With this subsample, instead of a dropout rate of 35% as found in the complete database of 409 patients, the study was conducted with a sample showing a dropout rate of only 18%. Therefore, there is a risk that the quantitative results are slightly biased and can only identify overall tendencies, but nevertheless complementing our findings from the qualitative data

## Conclusion

Rabies should be controlled using a One Health approach including vaccinating 70% of the dog population (39). However, in view of notoriously low dog vaccination coverage in many low- and middle-income countries, this study has tried to elucidate factors that would help to improve the situation for dog bite victims. Non-compliance with prophylactic treatment in vaccination centers in San-Pedro and throughout Côte d'Ivoire by persons exposed to rabies remains a real obstacle to the success of the national strategy for rabies control in Côte d'Ivoire. This study identified the key determinants of non-adherence to PEP in San Pedro by using a holistic perspective when looking into health care seeking practices. A combination of low detailed knowledge and therefore misjudging the severity of rabies as well as reliance on home care and traditional medicine in the context of low, unaffordable health care coverage emerged as the main factors for abandonment of PEP. Lack of knowledge highlights the importance of communication and awareness raising about rabies in the different socio-cultural groups of the communities.

These results show that many efforts need to be made to achieve the goal of eliminating rabies by 2030. It therefore appears necessary at the institutional level for health workers to strengthen and operationalize the monitoring and awareness system. By setting up a patient awareness and follow-up service that can widely explain to patients and guardians the dangers of attacks by dogs, cats and rodents, and that can contact them for appointments would contribute to raising awareness of the danger of rabies and the importance of patient care. It is also necessary for health officials to reinforce the training of community health workers on the dangers of dog bites to enable them to be “equipped” to better refer patients to the rabies vaccination center. Equipping the local rabies control committee set up in San-Pedro during the GAVI project to organize awareness campaigns by group of neighborhoods, by school and from local radio stations is also necessary. Another action to be implemented will be to integrate a rabies program in primary and secondary schools to improve children's knowledge of rabies, even though our study reveals their lack of decision-making power in their care. It is also essential to bring the center closer to the population by creating representations in two other sub-prefectures. New intradermal vaccination schedules with fewer doses promoted by WHO will allow to reduce indirect costs and are likely to increase completion rates.

## Data Availability Statement

The raw data supporting the conclusions of this article will be made available by the authors, without undue reservation.

## Ethics Statement

The study was reviewed and approved by the ethical approval of the GAVI Project (N/Ref: 072/MSHP/CNERkp) from the National Ethics Committee of Côte d'Ivoire. The agreement of the Northwest and Central Ethics Committee of Switzerland was also obtained [Ethics Committee of North Western and Central Switzerland (EKNZ) Basec 2016-00,220]. Oral informed consent was obtained by study participants.

## Author Contributions

RN'G: sociologist, carried out the study the general writing of the document. KH-T: social anthropologist and contributed to all stages of the project from conception to finalization. DA: socio-anthropologist, contributed to the academic supervision of the work, and contributed to the correction and development of data collection tools. ST: physician, her contribution consisted of providing guidance, and correcting the document. VK: veterinarian, guided this work, and contributed to the correction. AN: veterinarian, participated in the guidance of the work, and helped with the drafting of the manuscript. GN: medical entomologist and epidemiologist and contributed to the correction of the document. IK: professor of Sociology, supervised this work at the academic level, and contributed to the sociological orientation of this work and to the editing. KK: epidemiologist, contributed to statistical analysis, and the correction of the document. BB: Veterinary-epidemiologist and specialist in rabies control in Africa, the principal investigator of the project, and has contributed to all stages of the project. All authors contributed to the article and approved the submitted version.

## Funding

The research for this article was conducted under the DELTAS Africa Initiative (Africa One-ASPIRE/DEL-15-008). Africa One-ASPIRE is funded by a consortium of donors including the African Academy of Sciences (AAS), the Alliance for Accelerating Scientific Excellence in Africa (AESA), the New Partnership for Africa's Development (NEPAD) Planning and Coordinating Agency, the Wellcome Trust (107753/A/15/Z), and the British Government.

## Conflict of Interest

The authors declare that the research was conducted in the absence of any commercial or financial relationships that could be construed as a potential conflict of interest.

## Publisher's Note

All claims expressed in this article are solely those of the authors and do not necessarily represent those of their affiliated organizations, or those of the publisher, the editors and the reviewers. Any product that may be evaluated in this article, or claim that may be made by its manufacturer, is not guaranteed or endorsed by the publisher.
